# Trends and Developments in the Detection of Pathogens in Central Nervous System Infections: A Bibliometric Study

**DOI:** 10.3389/fcimb.2022.856845

**Published:** 2022-04-29

**Authors:** Yangyang Guo, Yanlin Yang, Ming Xu, Guangzhi Shi, Jianxin Zhou, Jindong Zhang, Hongliang Li

**Affiliations:** ^1^ Intensive Care Unit, Beijing Tiantan Hospital, Capital Medical University, Beijing, China; ^2^ Department of Gastroenterology, Peking University Third Hospital, Beijing, China

**Keywords:** central nervous system infections, pathogen detection, bibliometrics (source: MeSH NLM), CiteSpace, metagenomic next-generation sequencing (mNGS)

## Abstract

**Introduction:**

Rapid, sensitive, and specific laboratory assays are critical for the diagnosis and management of central nervous system (CNS) infections. The purpose of this study is to explore the intellectual landscape of research investigating methods for the detection of pathogens in patients with CNS infections and to identify the development trends and research frontier in this field.

**Methods:**

A bibliometric study is conducted by analyzing literature retrieved from the Web of Science (WoS) Core Collection Database for the years 2000 to 2021. CiteSpace software is used for bibliometric analysis and network visualization, including co-citation analysis of references, co-occurrence analysis of keywords, and cooperation network analysis of authors, institutions, and countries/regions.

**Results:**

A total of 2,282 publications are eventually screened, with an upward trend in the number of publications per year. The majority of papers are attributed to the disciplines of MICROBIOLOGY, INFECTIOUS DISEASES, IMMUNOLOGY, NEUROSCIENCES & NEUROLOGY, and VIROLOGY. The co-citation analysis of references shows that recent research has focused on the largest cluster “metagenomic next-generation sequencing”; the results of the analysis of the highest-cited publications and the citation burst of publications reveal that there is a strong interest stimulated in metagenomic next-generation sequencing. The co-occurrence analysis of keywords indicates that “infection”, “pathogen”, “diagnosis”, “gene”, “virus”, “polymerase chain reaction”, “cerebrospinal fluid”, “epidemiology”, and “metagenomic next-generation sequencing” are the main research priorities in the field of pathogen detection for CNS infections, and the keyword with the highest strength of burst is “metagenomic next-generation sequencing”. Collaborative network analysis reveals that the USA, the Centers for Disease Control and Prevention of USA, and XIN WANG and JENNIFER DIEN BARD are the most influential country, institution, and researchers, respectively.

**Conclusions:**

Exploring more advanced laboratory assays to improve the diagnostic accuracy of pathogens is essential for CNS infection research. Metagenomic next-generation sequencing is emerging as a novel useful unbiased approach for diagnosing infectious diseases of the CNS.

## 1 Introduction

Central nervous system (CNS) infections are inflammatory conditions of the brain and spinal cord caused by a variety of pathogenic microorganisms, including meningitis, encephalitis, myelitis, and abscesses. CNS infectious diseases are the main cause of morbidity and mortality worldwide (Tunkel et al., 2008; Venkatesan et al., 2013). For CNS infections, empiric antimicrobial therapy is usually suboptimal. Early detection of the causative organism in severely infected patients is essential to informing clinical intervention and the use of appropriate target antibiotics (Gu et al., 2021). Without appropriate treatment, it will progress rapidly and lead to secondary neurologic emergencies, particularly stroke, epilepsy, hydrocephalus, and intracranial hypertension (McEntire et al., 2021). Moreover, it can prolong hospital stays, increasing medical costs and the risk of death (Kanjilal et al., 2019). In addition, patients who remain undiagnosed will always require empiric broad-spectrum antibiotic therapy, which will increase the risk of adverse side effects and antimicrobial resistance (Llor and Bjerrum, 2014). Fortunately, infectious diseases are one of the few critical neurological conditions where complete or near-complete recovery can be achieved after treatment, as appropriate antimicrobial therapy can often significantly improve the prognosis (McEntire et al., 2021). Therefore, timely diagnosis, accurate differentiation of the underlying causative agent, and appropriate treatment are vitally important (Kanjilal et al., 2019).

CNS infections can be induced by a considerable number of infectious agents, mainly viruses and bacteria; cases of fungi and parasites are much less common (Vetter et al., 2020). To identify viable pathogens, clinicians must rely on the microbiological examination of clinical specimens, particularly cerebrospinal fluid (CSF) (Kanjilal et al., 2019). Non-specific CSF testing methods include CSF chemistry and cell counting, as well as Gram staining and culture. Pathogen-specific detection techniques include 1) pathogen-specific antibody reactions, which provide indirect evidence of infection; 2) direct detection of a pathogen by microscopic observation or direct detection of microbial antigens; and 3) direct detection of pathogen nucleic acids from prespecified targets by PCR. These tests are known as traditional diagnostic techniques, and they have their advantages and limitations (Kanjilal et al., 2019; Vetter et al., 2020). Isolation and culture of microorganisms indicate the presence of viable microorganisms but are less sensitive and time-consuming. Both PCR and serology require the clinician to select the pathogen being interrogated. These complexities often lead clinicians to utilize a patchwork of tests, which increases time consumption and financial costs (Ramachandran and Wilson, 2018; Kanjilal et al., 2019; Bharucha et al., 2019; Houlihan et al., 2019). There is therefore an urgent need to explore a more comprehensive, rapid, and accurate diagnostic approach to improve the efficiency of diagnosis of CNS infections.

Bibliometrics allows for the recognition of emerging trends and knowledge structures in the research field through a quantitative analysis of patterns in the scientific literature (Chen et al., 2012; Chen et al., 2020; Deng et al., 2020; Zhang et al., 2020). To provide an accurate, validated, and systematic overview of developments in pathogen detection technologies for CNS infections, to explore the latest detection methods and their effectiveness, and to identify thematic trends, research frontiers, and leading collaborations among researchers at various institutions, we conducted this bibliometric study by using CiteSpace software.

## 2 Materials and Methods

### 2.1 Source Database

In this study, we retrieved all literature data on pathogen detection in central nervous system infections indexed in the Web of Science (WoS) Core Collection. The literature search was performed on August 12, 2021. The WoS Core Collection database is often used for bibliometric analysis because it is continuously and dynamically updated and has a rigorous assessment of publications (Chen et al., 2012). The WoS-based literature analysis is conducted to obtain general information on the year of publication, journal, organization, author, and research area distribution.

### 2.2 Search Strategy

Concerning data collection, the following retrieval strategy was developed: “(TS = (“central nervous system infectio*” OR “CNS infectio*” OR “Brain Abscess*” OR encephalopyosis OR meningitis OR Encephalitis OR Meningoencephalitis OR Ventriculitis)) AND (TS = (pathogen* NEAR detect* OR pathogen* NEAR identif* OR pathogen* NEAR test* OR pathogen* NEAR exam* OR microorganism NEAR test* OR microorganism NEAR identif* OR microorganism NEAR detect* OR microorganism NEAR exam*))”. The language of publication was set to “English”; the literature category was “Article OR Review”; and the timespan was selected as January 1, 2000, to August 12, 2021. Using this search criterion, a total of 2,282 documents were identified, including 1,949 articles and 333 reviews. The “Full Record and Cited References” for these records were also extracted into the CiteSpace software in “Plain Text” format. Duplicate records were checked using the software’s native functions, and no duplicates were found. As a result, 2,282 documents were used as the final dataset.

### 2.3 Software Application

Scientific mapping is one of the main methods for exploring the field of bibliometrics, and the information visualization software of CiteSpace is one of the most popular tools for mapping scientific knowledge (Chen, 2017). The CiteSpace software was developed in 2004 by Professor Chaomei Chen of the Drexel University College of Computing and Informatics using Java language (Chen, 2004; Chen, 2006). The software supports several types of bibliometric studies, including co-citation analysis, co-occurrence analysis, and collaborative network analysis, ultimately visualizing the structure, regularity, and distribution of scientific knowledge (Chen and Song, 2019). By running the CiteSpace software, we used cluster analysis and burst keyword analysis to investigate research trends and hotspots in the field of CNS infection pathogen detection. We conducted a collaborative network analysis to study research collaborations between countries, institutions, and researchers.

### 2.4 Analysis Parameters

We used keywords and key references to predict research prospects and research hotspots. The parameters of CiteSpace were set as follows: time slicing (2000–2021), years per slice (Venkatesan et al., 2013), term source (all selection), node types (choose one at a time), links strength (Cosine), selection criteria (top 50 items), and pruning (pathfinder). The silhouette value implies the homogeneity of the cluster network and ranges from -1 to 1. The closer the value is to 1, the more homogeneous the network is. When the silhouette value is greater than 0.5, the clustering results are considered reasonable. When the silhouette value is greater than 0.7, the clustering results are considered to be highly reliable (Chen et al., 2012; Chen et al., 2019). The Q value of the clustering network indicates the modularity of the network, ranging from 0 to 1. The higher the value, the better the clustering results obtained by the network. When the modularity Q value is greater than 0.5, it indicates that the network clustering structure is significant (Chen et al., 2019).

## 3 Results

### 3.1 Analysis of Publications and Citation Counts

**Figure 1:**
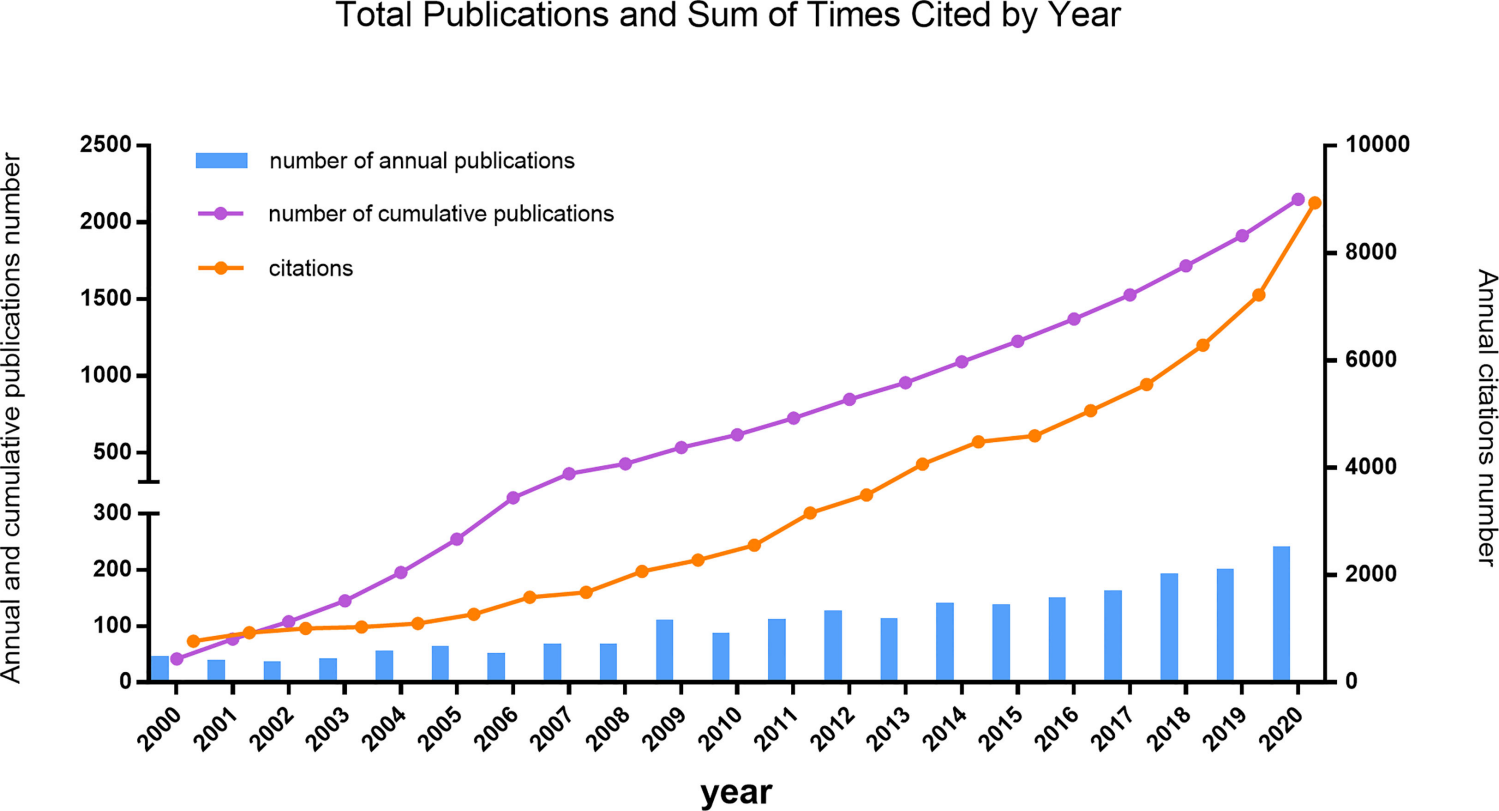
The number of annual and cumulative published articles, and the sum of annual citations related to pathogen detection for CNS infection research, from 2000 to 2020.

The input data for our project were generated by combining the results of multiple topic search queries on the Web of Science. The final dataset contains 2,282 bibliographic records of English-language articles or review types for the period January 1, 2000, to August 12, 2021. **Figure 1** shows the number of annual and cumulative publications and the sum of times cited by year from January 1, 2000, to December 31, 2020. Over the past 20-year period, the trajectory has shown two phases: an initial period (2000–2010) with a slow pace of development, and a period of growth (2011–2020). The number of publication outputs increased from 42 in 2000 to 236 in 2020, with an average of 56.3 in the period 2000–2010 and 153.1 in the period 2011–2020.

### 3.2 Disciplines and Topics Involved in the Detection of Pathogens of CNS Infections

Each article indexed by WoS has one or more subject categories. To reveal the disciplines involved in pathogen detection for CNS infections, we ran CiteSpace with “category” as the node type. **Supplementary Figure 1** shows the network of such subject categories after being simplified by Pathfinder network scaling, which retains the most prominent connections. The top-ranked category was MICROBIOLOGY, which had the largest circle with 540 citations. In second place is INFECTIOUS DISEASES with 443 citations. The third was IMMUNOLOGY, with citation counts of 283. The fourth- and fifth-ranked categories were NEUROSCIENCES & NEUROLOGY and VIROLOGY, with 258 and 244 citations, respectively. Meanwhile, the result showed that research on the detection of pathogens of CNS infections already encompasses multidisciplinary knowledge. Interdisciplinary collaboration between different fields may help to improve scientific work and maximize the potential.

### 3.3 The Intellectual Structure of Pathogen Detection in CNS Infections

**Figure 2:**
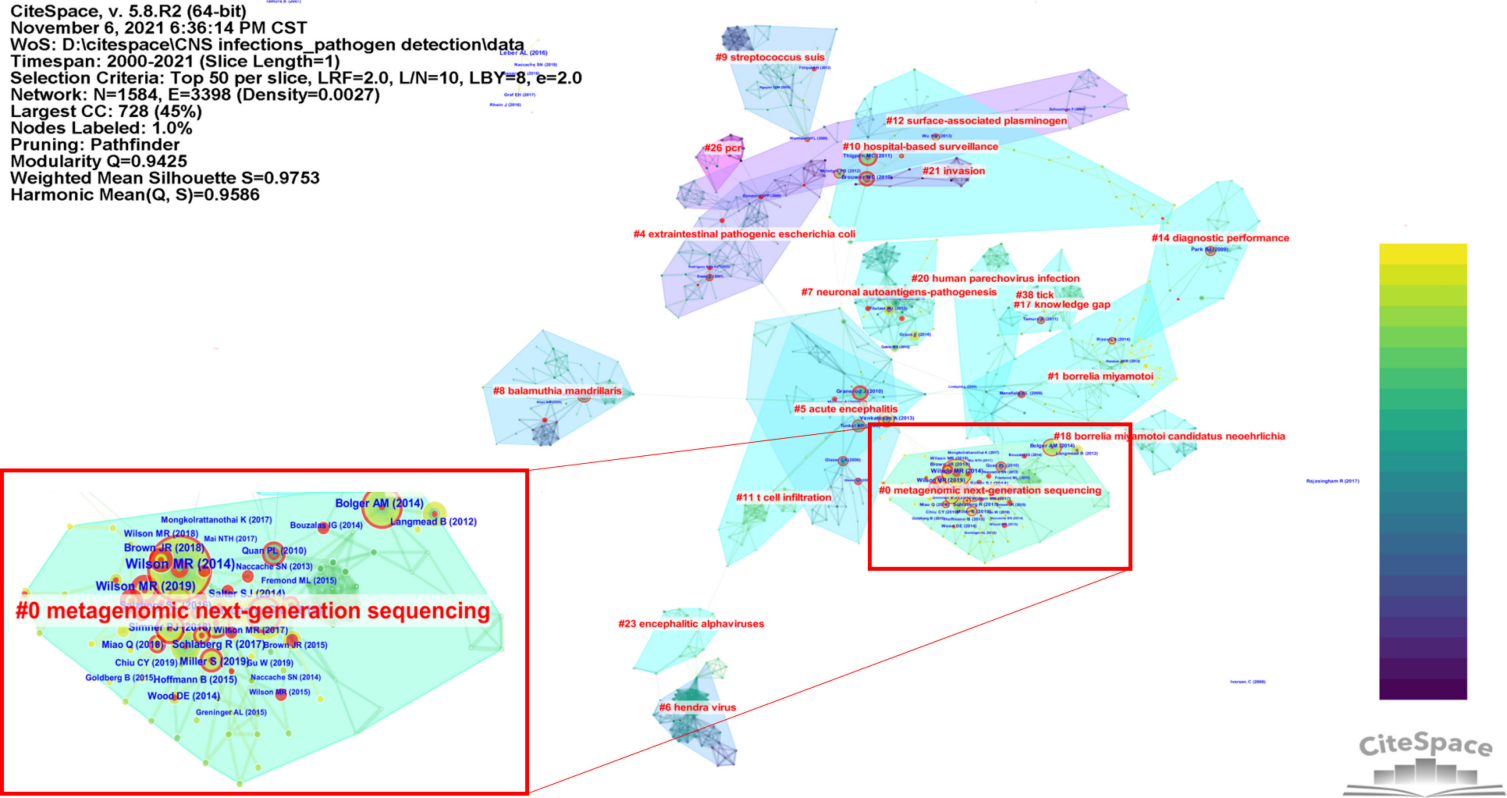
An overview of a network of co-cited references on pathogen detection in CNS infections.

**Figure 2** shows a cluster visualization of the reference co-citation network generated by CiteSpace software, based on publications from 2000 to 2021. The top 50 most cited publications for each year were used to construct a network of references cited in that year, and the individual networks were then synthesized. As a result, the co-citation networks were eventually divided into clusters of co-cited references, so that references were closely linked within the same cluster but loosely linked between different clusters. The clusters are referred to with labels selected by the log-likelihood ratio test method. The network has a modularity Q value of 0.9425, which is considered to be very high and indicates that the specialties in the scientific mapping are well defined in terms of co-citation clusters (Chen, 2017). The silhouette value of 0.9753 revealed the high reliability of the clustering results. The different-colored areas represent the time when the co-citation links for these areas first appeared. The purple areas were generated earlier than the blue areas, and the green areas were generated after the blue areas. Clusters are labeled in red text. The different nodes in the map represent cited references, with landmark references labeled in blue. Nodes with red tree rings are references with citation bursts, i.e., rapid growth in the number of citations.

**Table 1** shows details of the ten largest reference clusters in the co-citation network, with clusters listed by their size, i.e., the number of members in each cluster. Clusters with few members tend to be less representative than larger clusters, as the smaller clusters may be formed by the citing behavior of a few publications. All clusters in **Table 1** are highly homogeneous and have a high silhouette score, with clusters #0 and #8 having a silhouette score of 1. The average year of publication of a cluster indicates its recentness. For example, the most recently formed cluster, cluster #0 on metagenomic next-generation sequencing, has the average year of 2014.

**Table 1:** Summary of the largest 10 clusters.

Cluster ID	Size	Silhouette	Label (LLR)	Average year
0	94	1	Metagenomic next-generation sequencing	2014
1	67	0.938	*Borrelia miyamotoi*	2011
4	57	0.953	Extraintestinal pathogenic *Escherichia coli*	2004
5	56	0.975	Acute encephalitis	2008
6	55	0.976	*Hendra* virus	2006
7	54	0.992	Neuronal autoantigens—pathogenesis	2011
8	49	1	*Balamuthia mandrillaris*	2007
9	42	0.979	*Streptococcus suis*	2006
10	39	0.947	Hospital-based surveillance	2011
11	37	0.956	T-cell infiltration	2011

**Figure 3:**
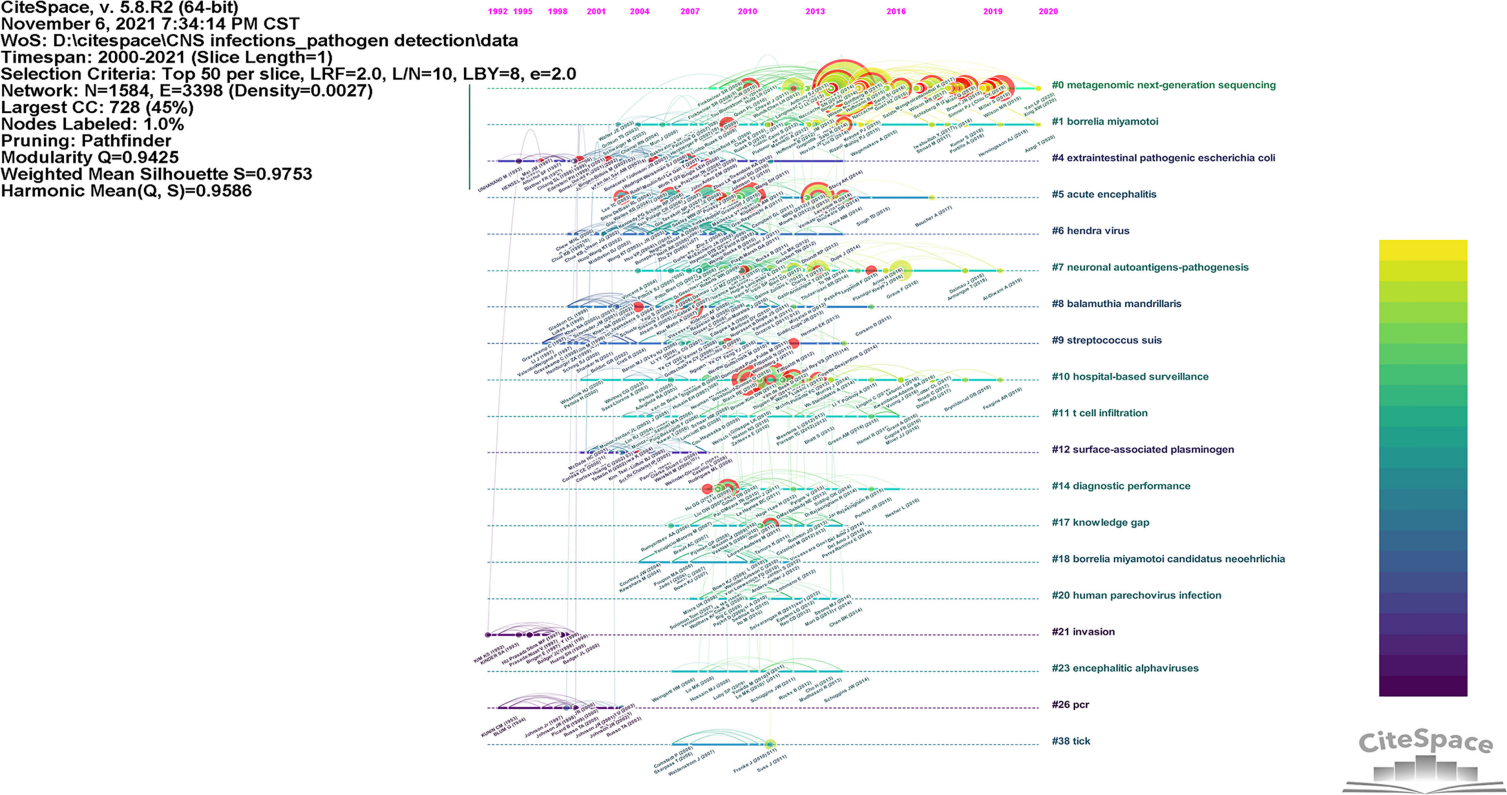
A timeline view of the co-citation clusters. The major clusters are labeled on the right.

**Figure 3** shows the co-citation clusters as a timeline view, which depicts the clusters along a horizontal timeline and is arranged vertically in descending order of their size. As the timeline overview shows, the sustainability of a specialty varies. A specialty may go through an initial conceptualization stage, a growth in research capabilities through the flourish of research tools, an expansion stage when researchers apply their methods to disciplinary areas beyond the original research problems, and finally a decline stage (Shneider, 2009). Some clusters have a long duration, while others have a relatively short duration. Colored curves represent the co-citation links added by the corresponding-colored year. Nodes of large size or those with red tree rings are of particular interest because they either are highly cited or have citation bursts or both.

The following tables list the key players in the four main clusters #0 (**Table 2**), #5 (**Table 3**), #7 (**Table 4**), and #10 (**Table 5**) to indicate their key research priorities.

**Table 2:** Cited references and citing articles of Cluster #0 on metagenomic next-generation sequencing.

Cited references		Citing articles	
Cites	Author (year) journal, volume, page	Coverage %	Author (year) title
60	Wilson et al. (2014) NEW ENGL J MED, V370, P2408	22	Forbes et al. (2018) Highlighting clinical metagenomics for enhanced diagnostic decision-making: a step towards wider implementation.
42	Naccache et al. (2015) CLIN INFECT DIS, V60, P919	22	Ivy et al. (2018) Direct detection and identification of prosthetic joint infection pathogens in synovial fluid by metagenomic shotgun sequencing.
34	Wilson et al. (2019) NEW ENGL J MED, V380, P2327	21	Han et al. (2019) Mngs in clinical microbiology laboratories: on the road to maturity.
31	Bolger et al. (2014) BIOINFORMATICS, V30, P2114	20	Simner et al. (2018) Understanding the promises and hurdles of metagenomic next-generation sequencing as a diagnostic tool for infectious diseases.
24	Simner et al. (2018) CLIN INFECT DIS, V66, P778	19	Vu et al. (2016) Novel human astroviruses: novel human diseases?
23	Salzberg et al. (2016) NEUROL-NEUROIMMUNOL, V3, Pe251	19	Cordey et al. (2016) Astrovirus mlb2, a new gastroenteric virus associated with meningitis and disseminated infection.
22	Schlaberg et al. (2017) ARCH PATHOL LAB MED, V141, P776	16	Li et al. (2021) Next-generation sequencing of cerebrospinal fluid for the diagnosis of unexplained central nervous system infections.
21	Salter et al. (2014) BMC BIOL, V12, P87	16	Simner et al. (2018) Development and optimization of metagenomic next-generation sequencing methods for cerebrospinal fluid diagnostics.
21	Brown et al. (2018) J INFECTION, V76, P225	15	Kanjilal et al. (2019) Diagnostic testing in central nervous system infection.
21	Miller et al. (2019) GENOME RES, V29, P831	15	Saylor et al. (2015) Acute encephalitis in the immunocompromised individual.

**Table 3:** Cited references and citing articles of cluster #5 on acute encephalitis.

Cited references		Citing articles	
Cites	Author (year) journal, volume, page	Coverage %	Author (year) title
27	Venkatesan et al. (2013), CLIN INFECT DIS, V57, P1114	14	Kramer et al. (2013) Viral encephalitis in the ICU.
27	Granerod et al. (2010), LANCET INFECT DIS, V10, P835	10	Yansouni et al. (2013) Rapid diagnostic tests for neurological infections in central Africa.
21	Tunkel et al. (2008), CLIN INFECT DIS, V47, P303	8	Zimmer et al. (2016) Central nervous system infections.
20	Glaser et al. (2006), CLIN INFECT DIS, V43, P1565	7	Saylor et al. (2015) Acute encephalitis in the immunocompromised individual.
13	Mailles and Stahl (2009), CLIN INFECT DIS, V49, P1838	7	Yao et al. (2009) Detection of human herpesvirus-6 in cerebrospinal fluid of patients with encephalitis.

**Table 4:** Cited references and citing articles of cluster #7 on neuronal autoantigens—pathogenesis.

Cited references		Citing articles	
Cites	Author (Year) Journal, Volume, Page	Coverage %	Author (Year) Title
20	Titulaer et al. (2013) LANCET NEUROL, V12, P157	28	Lancaster and Dalmau (2012) Neuronal autoantigens-pathogenesis, associated disorders and antibody testing.
19	Graus et al. (2016) LANCET NEUROL, V15, P391	25	Iorio and Lennon (2012) Neural antigen-specific autoimmune disorders.
11	Dalmau et al. (2008) LANCET NEUROL, V7, P1091	16	Wong-Kisiel et al. (2012) Autoimmune encephalopathies and epilepsies in children and teenagers.
11	Irani et al. (2010) BRAIN, V133, P2734	14	Ramanathan, *Sudarshini* (2014) Autoimmune encephalitis: recent updates and emerging challenges.
10	Gable et al. (2012) CLIN INFECT DIS, V54, P899	10	O’Toole et al. (2013) Paraneoplastic and autoimmune encephalopathies.

**Table 5:** Cited references and citing articles of cluster #10 on hospital-based surveillance.

Cited references		Citing articles	
Cites	Author (year) journal, volume, page	Coverage %	Author (year) title
32	Brouwer et al. (2010) CLIN MICROBIOL REV, V23, P467	7	Boula et al. (2019) Hospital-based surveillance provides insights into the etiology of pediatric bacterial meningitis in Yaounde, Cameroon, in the post-vaccine era.
30	Thigpen et al. (2011) NEW ENGL J MED, V364, P2016	7	Nhantumbo et al. (2015) Frequency of pathogenic paediatric bacterial meningitis in Mozambique: the critical role of multiplex real-time polymerase chain reaction to estimate the burden of disease.
15	McIntyre et al. (2012) LANCET, V380, P1703	7	Mbaeyi et al. (2019) Improving case-based meningitis surveillance in 5 countries in the meningitis belt of sub-Saharan Africa, 2015-2017.
14	Wu et al. (2013) BMC INFECT DIS, V13, P26	6	Paye et al. (2019) Implementation of case-based surveillance and real-time polymerase chain reaction to monitor bacterial meningitis pathogens in chad.
10	Wang et al. (2012) J CLIN MICROBIOL, V50, P702	6	Sanogo et al. (2019) A new sequence type of Neisseria meningitidis serogroup c associated with a 2016 meningitis outbreak in mali.

#### 3.3.1 Cluster #0—Metagenomic Next-Generation Sequencing

Cluster #0 is the largest and most recently formed cluster. It contains 94 references from 2008 to 2020, with an average publication year of 2014. The whole period of cluster #0 is filled with high-impact contributions—large citation tree rings and periods of citation bursts are indicated in red. We will focus specifically on cluster #0 to identify emerging trends in the detection of pathogens in CNS infections.

We selected the 10 most cited references and 10 citing articles in this cluster (**Table 2**). The most cited article in this cluster was a case report by Wilson et al., entitled “Actionable Diagnosis of Neuroleptospirosis by Next-Generation Sequencing” (Wilson et al., 2014). The authors reported on a 14-year-old boy with severe combined immunodeficiency who presented with fever and headache for 4 months and eventually developed hydrocephalus and status epilepticus. However, diagnostic workup, including brain biopsy, during the etiological diagnosis was unrevealing. Finally, unbiased next-generation sequencing of CSF identified 475 (0.016%) of 3,063,784 sequence reads corresponding to *Leptospira* infection. Following targeted antimicrobial treatment, the disease was effectively controlled. Their work represents an important milestone. They are the first to apply next-generation sequencing to the diagnosis of CNS infections in clinical practice. The second most cited reference is from Naccache et al. (2015), reporting the diagnosis of neuroinvasive astrovirus-infected encephalitis in a 42-year-old immunocompromised man by unbiased next-generation sequencing. Wilson et al. (2019) conducted the first multicenter, prospective study to investigate the usefulness of metagenomic NGS of CSF for diagnosing inpatient usefulness of infectious meningitis and encephalitis. In the study, metagenomic NGS of CSF obtained from patients with meningitis or encephalitis improved the diagnosis of neurologic infections and provided actionable information in some cases.

The 10 selected citing articles cited 15%–22% of the 94 co-cited references in cluster #0. The most recently published one was written by Li et al. (2021). They conducted a comprehensive PubMed search of articles published from January 1, 2008, to June 26, 2020, to retrieve all available next-generation sequencing studies for the etiology of unexplained CNS infections in pediatric patients. They concluded that NGS could be useful in identifying the etiology of unexplained encephalitis, meningoencephalitis, and meningitis in pediatric patients, although the diagnostic value of NGS is difficult to quantify.

#### 3.3.2 Cluster #5—Acute Encephalitis

Encephalitis is a long-standing research hotspot because of its high mortality and morbidity rates and its complex etiology. This cluster of literature is concerned with the identification of the etiology, diagnosis, and management of encephalitis.

This cluster contains numerous nodes with red-ringed citation bursts. The first and third most cited articles are Venkatesan et al. (2013) and Tunkel et al. (2008), both of which are practice guidelines on encephalitis. Venkatesan et al. (2013) is a consensus document put forward by the International Encephalitis Consortium, a committee formed in 2010 with members across the globe. It proposes standardized case definition and diagnostic guidelines for the assessment of adults and children with suspected encephalitis. Tunkel et al. (2008) is a clinical practice guideline from the Infectious Diseases Society of America focused on the diagnosis and treatment of patients with encephalitis. Granerod et al. (2010) reported the results of a multicenter, population-based prospective study aimed at identifying the causes of encephalitis in patients in England and identifying clinical differences between causes. Moreover, the authors emphasized the importance of identifying the cause of encephalitis.

#### 3.3.3 Cluster #7—Neuronal Autoantigens—Pathogenesis

Over the last decade, an increasing number of cases of non-infectious, mainly autoimmune, encephalitis have been identified. This newly identified autoimmune encephalitis may be associated with antibodies against neuronal cell-surface or synaptic proteins and can develop with core symptoms similar to infectious encephalitis with neurological and psychiatric manifestations but without fever or CSF pleocytosis.

Dalmau et al. (2008) described the clinical features of patients with anti-NMDA receptor (NMDAR) encephalitis, usually presenting with acute behavioral changes, psychosis, and catatonia, and progressing to disorders including seizures, memory deficits, movement disorders, speech problems, and autonomic and respiratory disorders. Improvement in symptoms is associated with a decrease in serum antibody titers. Titulaer et al. (2013) conducted an observational cohort study to assess the presentation, symptom profile, immunotherapies used, timing of improvement, and long-term outcomes in anti-NMDA receptor (NMDAR) encephalitis. The findings provide evidence that immunotherapy and tumor resection are useful against NMDAR encephalitis.

#### 3.3.4 Cluster #10—Hospital-Based Surveillance

Hospital-based meningitis surveillance is an important method for studying the pathogenesis and molecular epidemiology of meningitis. It provides evidence for assessing epidemiological trends, guiding empirical antimicrobial and adjunctive therapy in hospitals, evaluating available laboratory methods for diagnosis, and providing evidence for future vaccination strategies.

A review published by Brouwer et al. (2010) describes the changing epidemiology of bacterial meningitis in the USA, reviewing global changes in pathogens followed by specific microbiological data on the impact of the development and widespread use of conjugate vaccines. They reviewed the available laboratory tests used to diagnose bacterial meningitis and provided recommendations for empiric antibiotic therapy. Accurate identification of pathogens is essential for the treatment of meningitis. Wu et al. (2013) evaluated the accuracy of real-time PCR, Gram staining, and culture for the diagnosis of *Streptococcus pneumonia*, *Neisseria meningitides*, and *Haemophilus influenza* meningitis. They concluded that real-time PCR and Gram staining were highly accurate in the diagnosis of meningitis caused by *S. pneumonia*, *N. meningitides*, and *H. influenza*. Also, real-time PCR and Gram staining were less affected by the presence of antibiotics and may be useful when antibiotics have been used previously.

### 3.4 Most Cited Articles

Due to their pioneering contributions, the most cited articles are often considered landmarks. **Supplementary Figure 2** shows the clustering of highly cited articles, and **Table 6** summarizes the top 10 highly cited articles. Cluster #0 has four articles in the top 10 landmark articles. Clusters #10 and #5 each have two articles. Ranked first by the number of citations is Wilson et al. (2014) in cluster #0 with 60 citations, followed by Leber et al. (2016) with 55 citations. The third-, fourth-, and sixth-ranked articles were all from cluster #0, namely, Naccache et al. (2015), Wilson et al. (2019), and Bolger et al. (2014), indicating that researchers are stimulating strong interest in metagenomic next-generation sequencing.

**Table 6:** Top 10 highest cited publications on pathogen detection for CNS infections.

Citation counts	References	DOI	Cluster ID
60	Wilson et al., 2014, NEW ENGL J MED, V370, P2408	10.1056/NEJMoa1401268	0
55	Leber et al., 2016, J CLIN MICROBIOL, V54, P2251	10.1128/JCM.00730-16	13
42	Naccache et al., 2015, CLIN INFECT DIS, V60, P919	10.1093/cid/ciu912	0
34	Wilson et al., 2019, NEW ENGL J MED, V380, P2327	10.1056/NEJMoa1803396	0
32	Brouwer et al., 2010, CLIN MICROBIOL REV, V23, P467	10.1128/CMR.00070-09	10
31	Bolger et al., 2014, BIOINFORMATICS, V30, P2114	10.1093/bioinformatics/btu170	0
30	Thigpen et al., 2011, NEW ENGL J MED, V364, P2016	10.1056/NEJMoa1005384	10
27	Venkatesan et al., 2013, CLIN INFECT DIS, V57, P1114	10.1093/cid/cit458	5
27	Park et al., 2009, AIDS, V23, P525	10.1097/QAD.0b013e328322ffac	14
27	Granerod et al., 2010, LANCET INFECT DIS, V10, P835	10.1016/S1473-3099(10)70222-X	5

### 3.5 Citation Bursts

**Figure 4:**
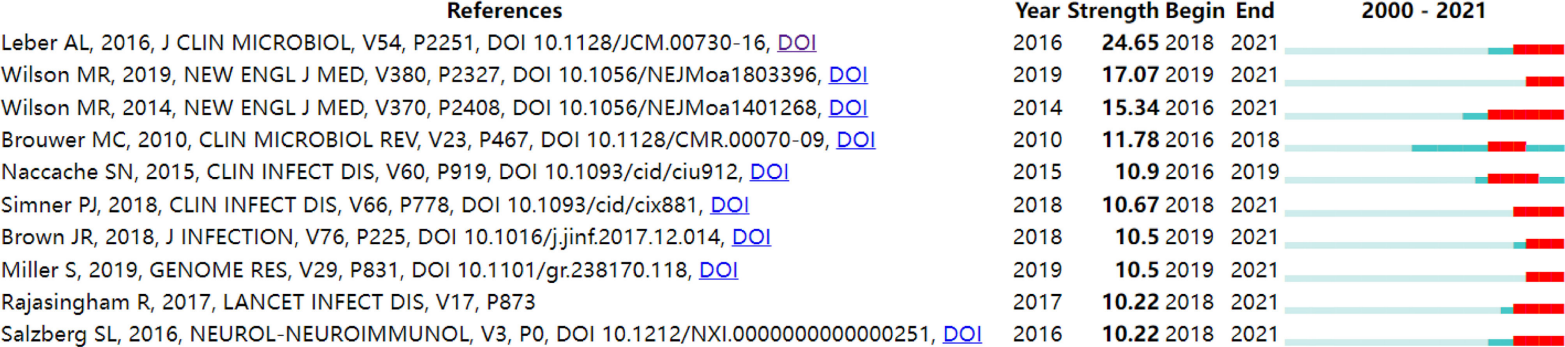
Top 10 articles with citation burst of publications on pathogen detection in CNS infections (sorted by strengths of burst).

Citation bursts have two attributes: the intensity and duration of the burst. Citation burst detection reveals abrupt changes in terms of citations over a specified period, thus identifying emerging research trends (Chen et al., 2012). **Figure 4** lists the references with the strongest citation bursts across the dataset for the period 2000–2021. Of the top 10 references with the strongest citation bursts, the second-, third-, fifth- to eighth-, and tenth-ranked articles are all from cluster #0 on metagenomic next-generation sequencing.

The top-ranked item by bursts was Leber et al. (2016) with bursts of 24.65. Leber et al. evaluated the performance of the FilmArray Meningitis/Encephalitis Panel compared to culture (bacterial analytes) and PCR (all other analytes) using 1,560 prospectively collected CSF specimens. The FilmArray Meningitis/Encephalitis (ME) Panel is a multiplexed *in vitro* diagnostic test for the simultaneous and rapid detection of 14 pathogens (*Escherichia coli K1*, *Haemophilus influenza*, *Listeria monocytogenes*, *Neisseria meningitides*, *Streptococcus pneumonia*, *Streptococcus agalactia*, *cytomegalovirus*, *enterovirus*, *herpes simplex virus 1 and 2*, *human herpesvirus 6*, *human parechovirus*, *varicella-zoster virus*, and *Cryptococcus neoformans*/*Cryptococcus gattii*) directly from CSF specimens. In conclusion, they concluded that the FilmArray ME Panel is a sensitive and specific test that can help diagnose meningitis/encephalitis. By using this comprehensive and rapid test, it is expected to improve patient outcomes and antimicrobial stewardship.

### 3.6 Keywords Co-Occurrence as Indicators of Research Hotspots

**Figure 5:**
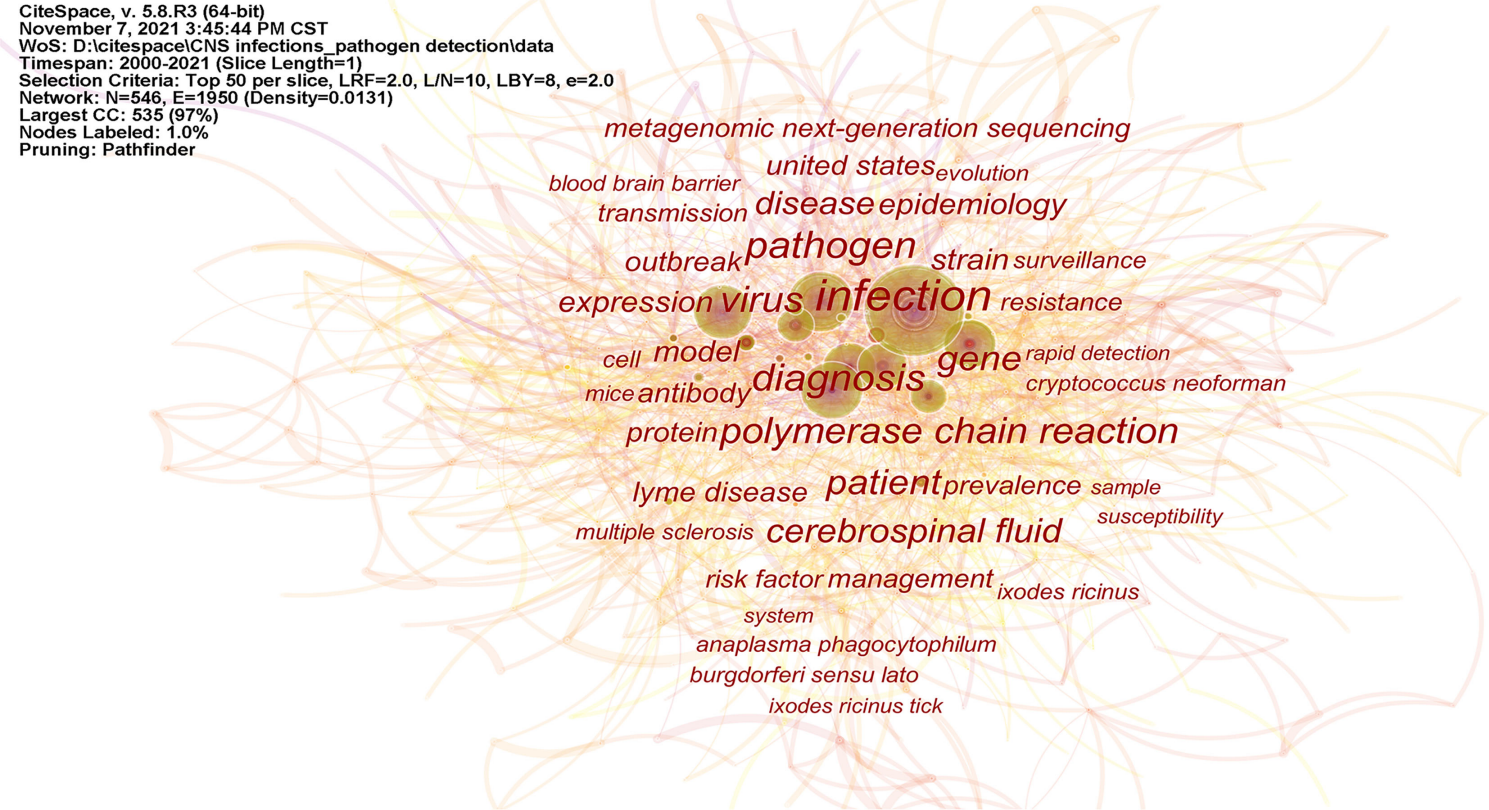
The network organized by co-occurrence keywords.

Keywords provide an accurate picture of what is hot in research at a given time and can be used to get a clear picture of the state of research. **Figure 5** shows the keywords with high co-occurrence rates. The larger the character, the more frequently the keyword appears. “Infection”, “pathogen”, “diagnosis”, “gene”, “virus”, “polymerase chain reaction”, “cerebrospinal fluid”, “epidemiology”, and “metagenomic next-generation sequencing” are the main research priorities in this field.

### 3.7 Analysis of Burst Keywords

**Figure 6:**
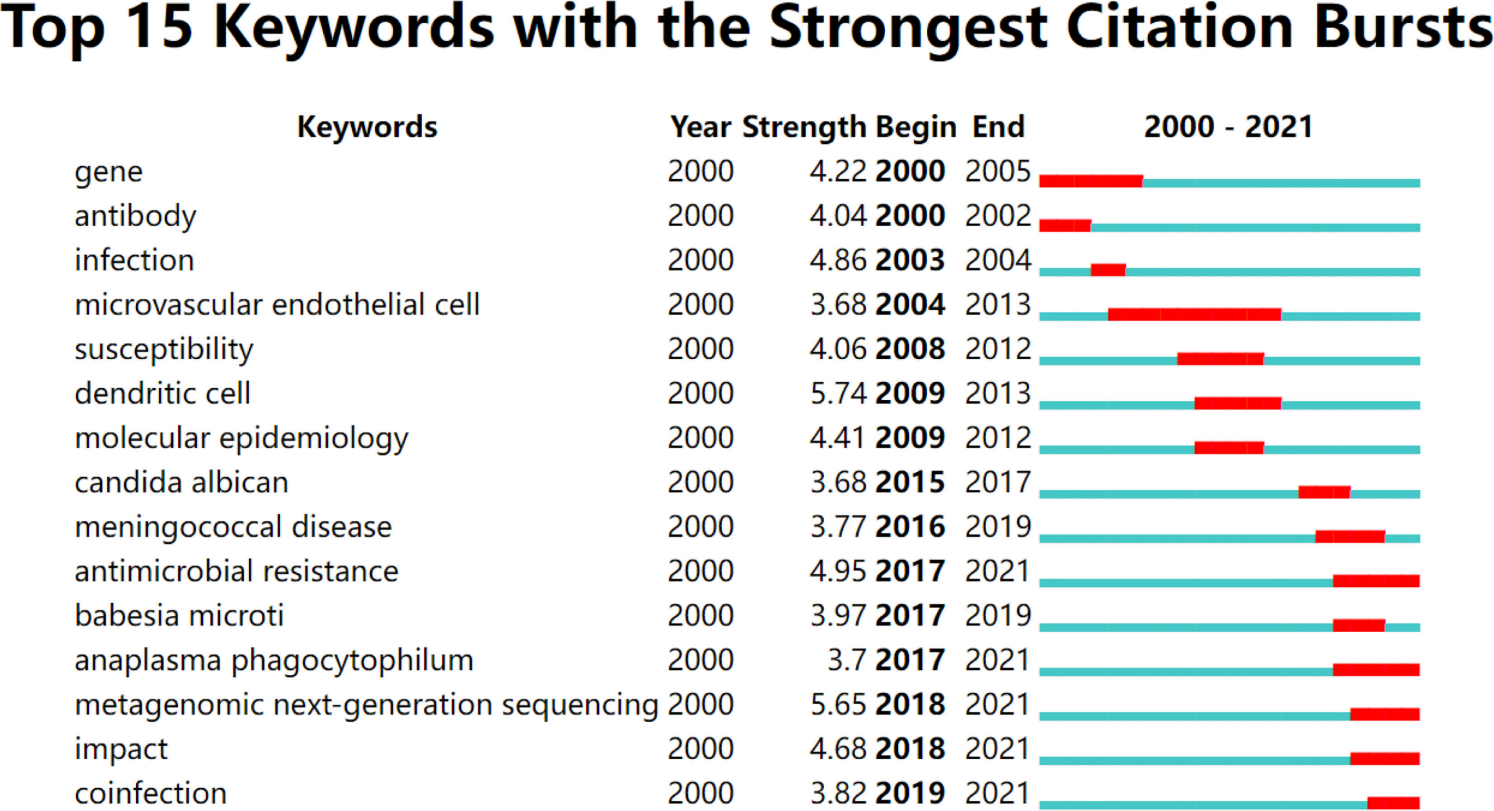
Top 15 keywords with burst impact (sorted by the beginning year of burst).

“Burst keywords” represent words that have been frequently cited over some time, and identifying burst keywords among all keywords may help to predict new frontier topics or research trends in the future. As we can see in **Figure 6**, the latest burst keywords are “antimicrobial resistance” (2017–2021), “anaplasma phagocytophilum” (2017–2021), “metagenomic next-generation sequencing” (2018–2021), “impact” (2018–2021), and “coinfection” (2019–2021). Of these five keywords, “metagenomic next-generation sequencing” had the highest strength of burst with a value of 5.65, indicating that it is a research frontier in the field of detection of pathogens of CNS infections.

### 3.8 Cooperation Network Among Countries

**Figure 7:**
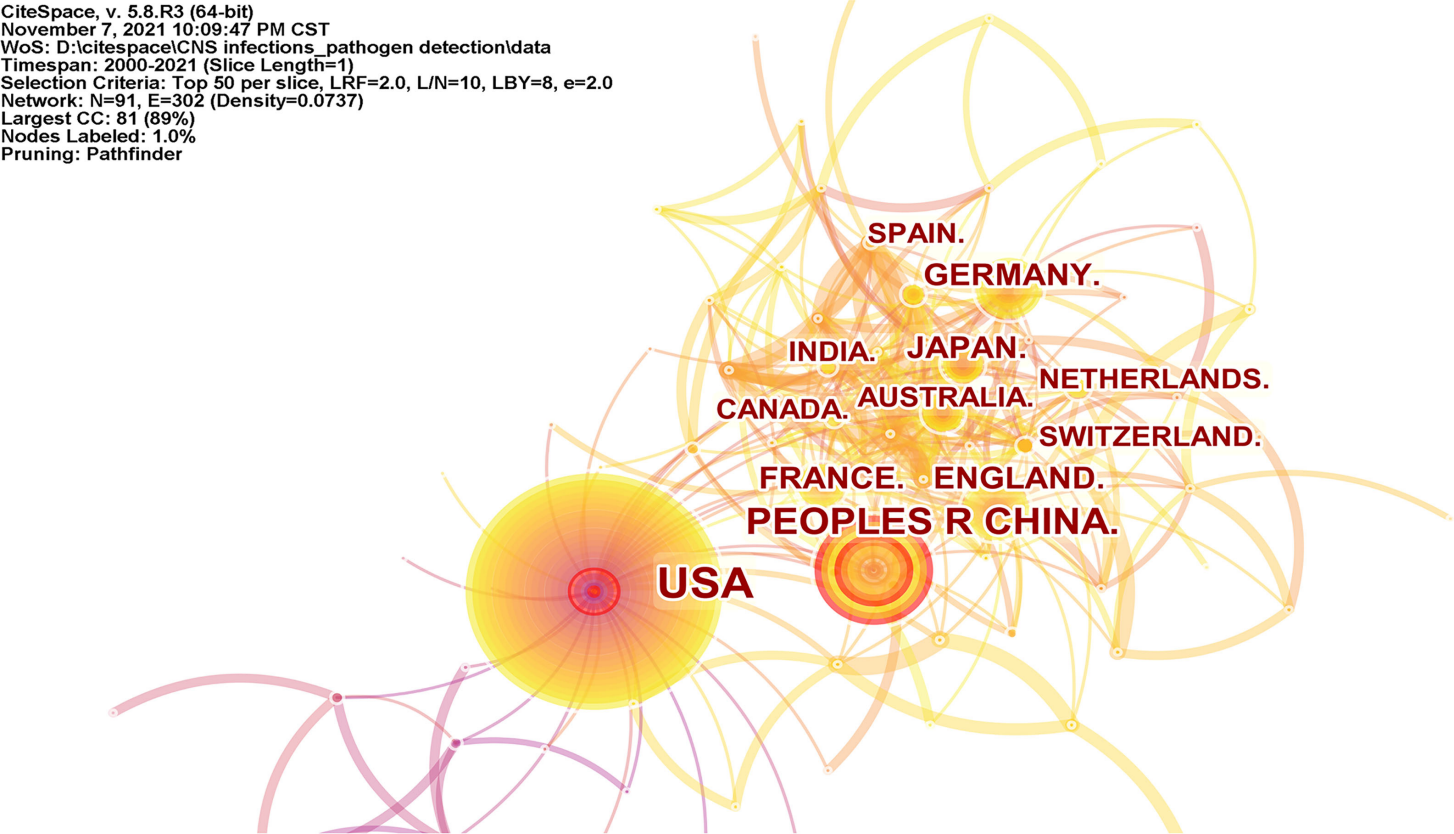
Visualization map of the scientific collaboration network analysis among countries/regions.

In the field of pathogen detection for CNS infections, 91 countries/regions have published relevant papers. The top 10 countries/regions by the number of publications are listed in **Supplementary Table 1**. In order of number of publications, the top 5 countries/regions were the USA (n = 769), the People’s Republic of China (n = 309), England (n = 146), Germany (n = 135), and France (n = 102). The network of cooperation between countries/regions is shown in **Figure 7**, with 91 nodes and 302 link lines. The nodes and the link lines between them represent the countries/regions and their collaborations, respectively. The larger the nodes, the more publications there are. The wider the line, the stronger the relationship. In this network map, the nodes in the USA, the People’s Republic of China, England, Germany, and France are larger and represent more publications. There are many positive collaborations between different countries/regions.

### 3.9 Cooperation Network Among Institutions

**Figure 8:**
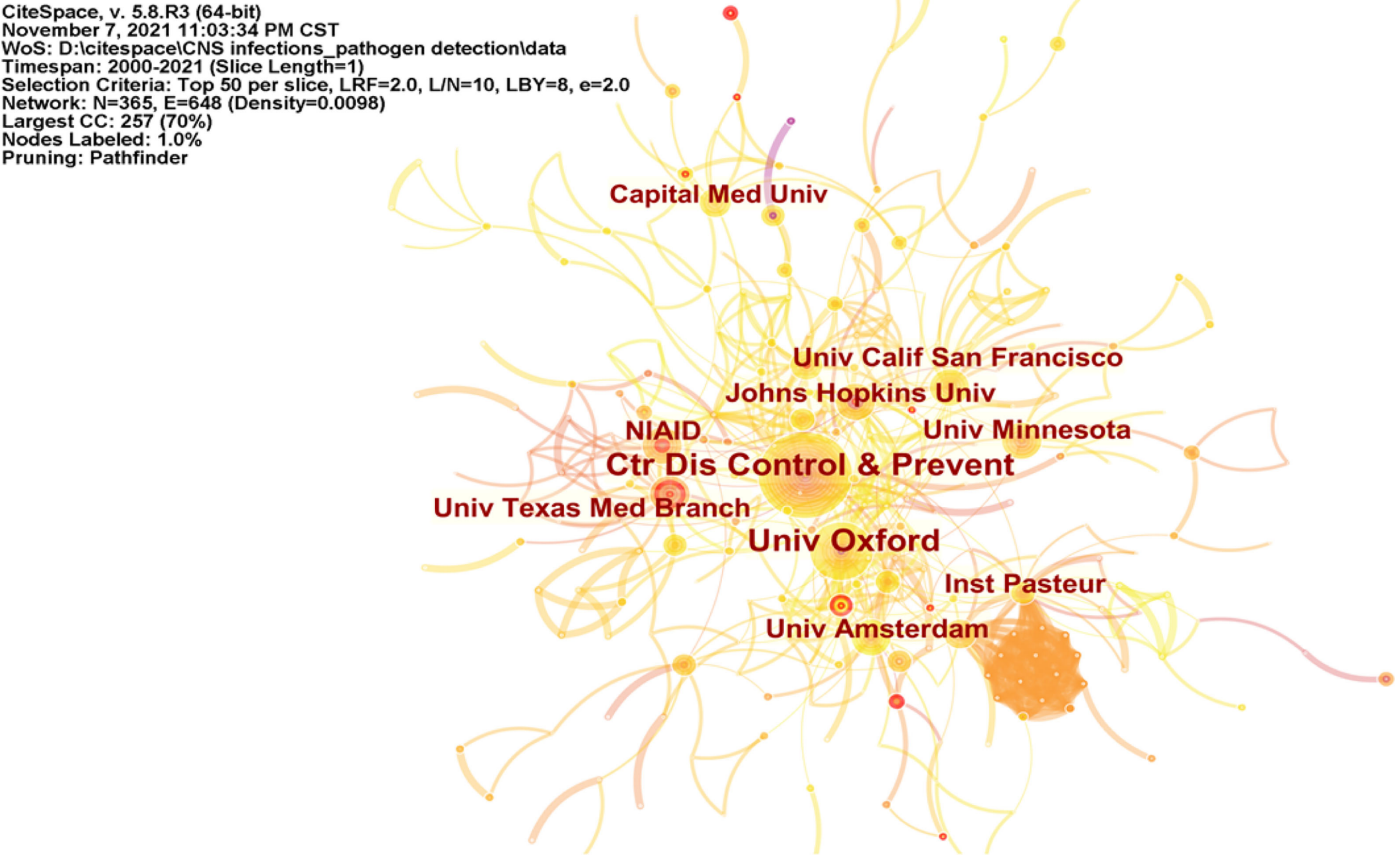
Visualization map of the scientific collaboration network analysis among institutions.

From 2000 to 2021, a total of 365 institutions have published in this field. **Supplementary Table 2** illustrates the top ten institutions by the number of publications, three-fifths of which are in the United States. The top five institutions contributing most to the field were the Centers for Disease Control and Prevention of USA, Oxford University, University of California, San Francisco, Johns Hopkins University, and the Pasteur Institute. The generated institutional network map identified 365 nodes and 648 link lines, representing institutions and their collaborative relationships; thus, active collaboration between different institutions was noted (**Figure 8**).

### 3.10 Cooperation Network Among Researchers

**Figure 9:**
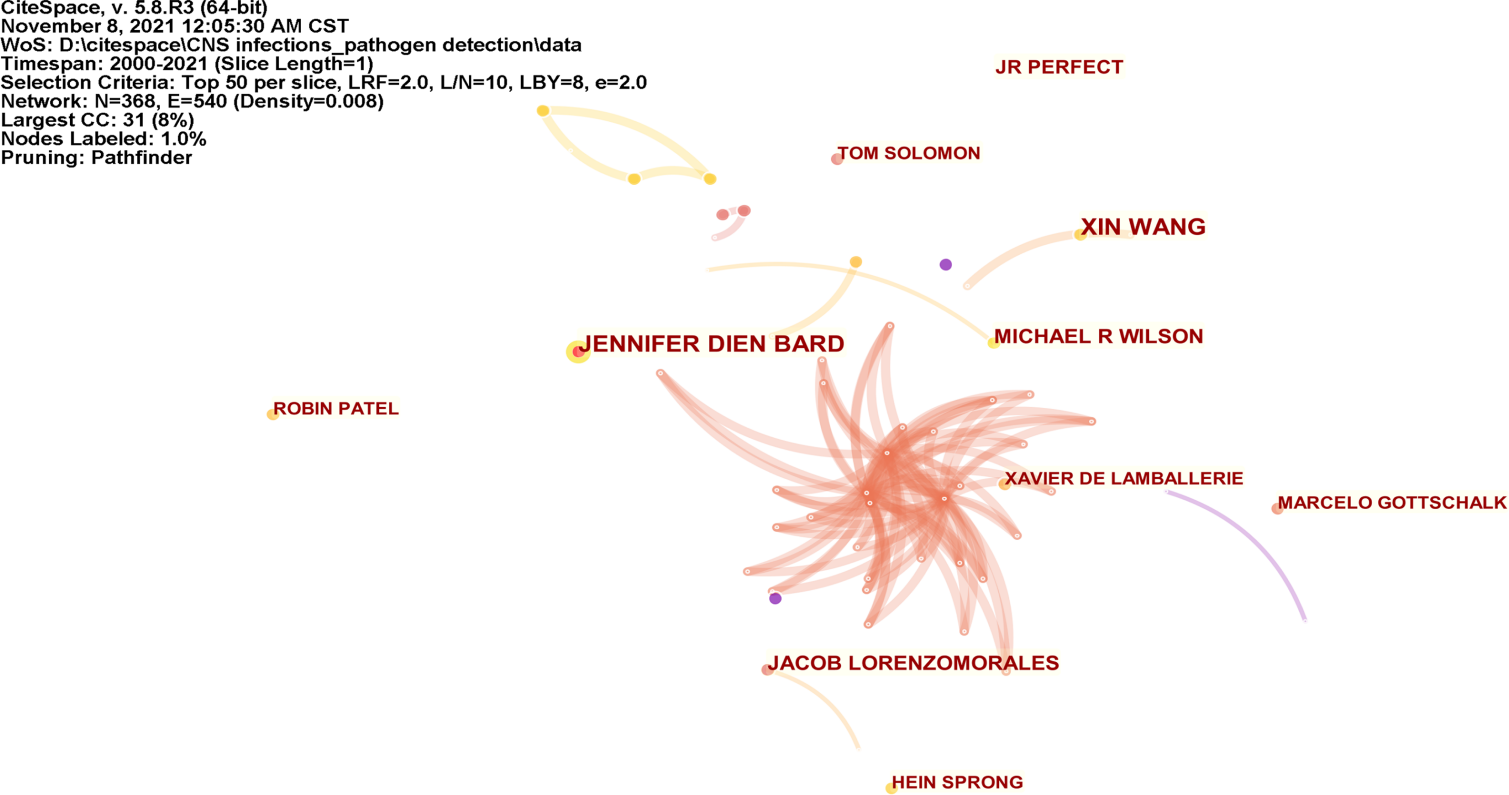
Visualization map of the scientific collaboration network analysis among researchers.

A total of 368 authors have published in the field in the last 20 years. **Supplementary Table 3** lists the top 10 authors in order of the number of publications. XIN WANG and JENNIFER DIEN BARD have published the most papers (n = 9) and are ranked first, followed By MICHAEL R WILSON and JACOB LORENZOMORALES (n = 7). The coauthorship network diagram is shown in **Figure 9** and contains 368 nodes and 540 collaboration lines. The nodes and the link lines between them represent the authors and their collaborations, respectively. As the figure shows, an international collaboration between top researchers was inadequate.

## 4 Discussion

In central nervous system infections, timely identification of the pathogen is key to effective treatment, but this remains a formidable challenge. Current data show that even in centers with the best resources, up to two-thirds of CNS infection cases remain undiagnosed (Granerod et al., 2010; McGill et al., 2018). Because most infectious syndromes have indistinguishable clinical presentations, there is an urgent need for broad-based multiplex diagnostic tests, which are currently unavailable for the vast majority of potential pathogens. Some microorganisms are difficult or impossible to culture, while others take weeks to culture and speciate (Rea et al., 2019). Accurate molecular tests by PCR and serological tests that detect pathogen-specific antibodies offer diagnostic alternatives to culture, but they are hypothesis-driven and require *a priori* suspicion of pathogenic pathogens (Kanjilal et al., 2019). Large multiplex test panels represent a paradigm shift in medical microbiology and clinical infectious diseases. The main benefit of these panels is the potential for more rapid results. However, because of the potential for contamination and the ability to detect latent or reactivated viruses, the results of the panels must be scrutinized and the laboratory must closely monitor the positivity rate (Hanson, 2016). Therefore, the development of more rapid, comprehensive, hypothesis-free pathogen detection methods with high sensitivity and specificity to improve diagnosis is a priority in neurological infection research.

In this study, we used CiteSpace software to explore trends and developments in the field of CNS infection pathogen detection research from 2000 to 2021. Trends in the number of annual publications and total citations of annual publications in the field reflect the interest and focus on the field over the years. The research area covers a wide range of disciplines and topics, including microbiology, infectious diseases, immunology, and neuroscience, reflecting the combined efforts of experts and scholars from various disciplines in the field. Collaborative network analysis can provide detailed information for assessing research collaborations and identifying key collaborators. Among countries/regions and institutions, the United States has an advantage in this area of research, given that it has published the most articles in this field and that three-fifths of the top ten institutions by the number of publications are located in the United States. Despite the close international scientific collaboration between scientists in this area of research, we found from the collaborative authorship network map that collaboration between top researchers is not sufficient.

Combining reference co-citation analysis and keyword co-occurrence analysis, this study found that research in the field of pathogenic diagnosis of CNS infections is focused on exploring new etiologies (autoimmune and rare microbial pathogens) and more advanced laboratory diagnostic methods. Based on the analysis of co-citation clusters, most cited articles, citation bursts, keyword co-occurrences, and burst keywords, metagenomic next-generation sequencing has become the most popular research hotspot in recent years.

Molecular diagnostic techniques have been continuously improved and developed in recent decades. The advent of high-throughput sequencing has made it possible to discover new microorganisms, rapidly address the causes of infectious disease outbreaks, and develop metagenomics. Metagenomics next-generation sequencing (mNGS) was initially limited to dedicated laboratories because of the high cost of instrumentation and consumables, as well as the need for specially trained personnel (Lipkin, 2013). With many published case reports and clinical study results confirming the validity of mNGS, and the continued decline in sequencing cost and time, mNGS is increasingly being used for clinical diagnoses, providing clinicians with a powerful diagnostic tool that has greatly improved our ability to detect infectious disease pathogens in clinical samples (Goldberg et al., 2015; Allcock et al., 2017).

Metagenomic next-generation sequencing is a comprehensive and rapid diagnostic tool. It allows unbiased and detailed testing of the total DNA or RNA content of all currently known pathogenic microorganisms (Fei et al., 2020). It offers a powerful advantage in infectious disease diagnosis as it does not require special probes and targeting primers and facilitates rapid detection of pathogens, including non-culturable organisms and novel organisms (Liu et al., 2015; Perlejewski et al., 2016; Bukowska-Ośko et al., 2017). mNGS also offers a diagnostic advantage over conventional methods for patients who have received empirical antimicrobial therapy before sample collection. The empirical use of antibiotics significantly reduces the detection rate of conventional methods by approximately 20%, whereas the detection rate of mNGS is unaffected (Zhang et al., 2020). There is growing evidence that mNGS plays an essential role in the diagnostic workup of CNS infections. Potentially treatable pathogens, such as *Leptospira*, *Brucella*, and *Balamuthia mandrillaris*, and non-treatable pathogens, such as *astroviruses* and novel viruses, which were not clinically suspected, have been identified using this technique (Wilson et al., 2014; Naccache et al., 2015; Greninger et al., 2015; Mongkolrattanothai et al., 2017). In 2017, the French “Guidelines on the management of infectious encephalitis in adults” recommended mNGS as level 1 evidence to assist clinical decision-making in CNS infections (Stahl et al., 2017).

Despite the appeal of mNGS for infectious disease diagnosis, there are many challenges before the technology becomes mainstream and part of the standard of clinical care. 1) Economic costs: given the cost of analysis and the required informatics infrastructure (e.g., sequencing platforms, computing resources, data storage), the use of mNGS is currently still limited to a small number of diagnostic laboratories (Zanella et al., 2019). 2) Standardization: standardized operational procedures for NGS diagnostics, including clinical specimen collection, sequencing parameters, data analysis algorithms, reference databases, quality control, and data reporting, need to be further defined (Goldberg et al., 2015). 3) Technical challenge: there is still a lack of effective methods to reduce host contamination and improve the sensitivity of mNGS to clinical CSF samples. Human DNA contamination is a major challenge for mNGS in detecting pathogens in most clinical specimens. Elevated white blood cell counts are one of the main manifestations of infection and a major source of host contamination. This is because the high background of host DNA greatly reduces the sequence coverage of pathogens, thereby reducing the sensitivity of mNGS to detect pathogens (Hasan et al., 2016). Several attempts have been made to reduce host DNA contamination. Selective degradation of host DNA in blood samples with methylation-dependent endonucleases, based on the different methylation patterns between host DNA and pathogen genomes, results in an enrichment of pathogen DNA (Auburn et al., 2011; Oyola et al., 2013). However, this approach does not apply to all pathogens, as many types of microorganisms also have methylated CpGs (e.g., fungi). Another approach to selectively isolate host DNA is to hybridize to pathogen DNA using specific probes complementary to the pathogen genome and capture it for amplification and sequencing. Although these targeted enrichment methods can partially decontaminate the human genome, they are insufficient to provide comprehensive, unbiased detection of infections of unknown etiology (Melnikov et al., 2011; Bright et al., 2012; Smith et al., 2012). 4) Interpret test results with caution: although one of the advantages of mNGS analysis is the ability to detect DNA or RNA of many microorganisms directly from patient samples, a positive test result does not establish that it is from a live microorganism, whereas a positive culture result indicates the presence of a live organism. In addition, mNGS has particular limitations in distinguishing which organisms are colonized, conditionally pathogenic, or active pathogens (Goldberg et al., 2015). 5) Higher demands on clinicians: molecular diagnostics presents challenges in terms of data interpretation and reporting, and the shift to molecular diagnostics will require a change in clinician thinking. Upcoming generations of physicians will need to adapt to the strengths and limitations of these new tools (Goldberg et al., 2015).

Therefore, we need to take additional efforts to address these technical challenges and to further reduce the economic costs. At the same time, we should also recognize that, in addition to identifying pathogens, treatment is vital. Therefore, the identification of virulence genes and sequence variants of causative pathogens, assessment of antibiotic resistance, and evaluation of the effectiveness of antibiotic therapy should also be given high priority (Goldberg et al., 2015). More clinical studies are needed to explore the capabilities of mNGS in these applications (Goldberg et al., 2015).

## 5 Conclusion

In conclusion, the analysis and citation-based expansion of the literature on pathogen detection in CNS infections have outlined the trajectory of the evolution of collective knowledge over the past two decades and highlighted areas of active pursuit. The exploration of more advanced laboratory tests to identify pathogenic pathogens has always been the core of research priorities in neurological infections. As a comprehensive, rapid, and unbiased test, mNGS offers diagnostic advantages over traditional methods, but at the same time, mNGS still has several technical and economic limitations that need to be improved upon. The results of our review and analysis of the hotspots and research trends may promote the development of this field.

## 6 Limitations

There are some limitations to this study that need to be noted and addressed. Firstly, as our study was based on references searched in the WoS Core Collection database, this means that we may have omitted some important studies in other medical databases. However, because different databases have different methods of calculating citation frequencies, it is not appropriate to merge data from different databases, and it is difficult for current bibliometric software to merge two and more databases. Therefore, previous studies have usually selected one database as the primary search database (Zhong et al., 2020; Chen et al., 2020; Wu et al., 2021). Among these, WoS Core Collection is a commonly used reference database in bibliometric research. Secondly, our study only analyzed the English language literature, which may have led to the incompleteness of the data and the deviation of the research results. Thirdly, the deadline for the research publications in this study was August 12, 2021. All data in this work do not fully reflect the reality of 2021 and could be a reference.

## Data Availability Statement

The original contributions presented in the study are included in the article/**Supplementary Material**. Further inquiries can be directed to the corresponding authors.

## Author Contributions

HL and YG designed the study. YG and JdZ performed the search. YG, JdZ, and MX analyzed the data and performed the software analysis. YG drafted the first vision. GS, JxZ, and HLL revised the manuscript critically. All authors contributed to the article and approved the submitted version.

## Funding

This work was supported by the Beijing Tiantan Hospital Youth Fund (2018-YQN-15) and the National Natural Science Foundation of China (82000510).

## Conflict of Interest

The authors declare that the research was conducted in the absence of any commercial or financial relationships that could be construed as a potential conflict of interest.

## Publisher’s Note

All claims expressed in this article are solely those of the authors and do not necessarily represent those of their affiliated organizations, or those of the publisher, the editors and the reviewers. Any product that may be evaluated in this article, or claim that may be made by its manufacturer, is not guaranteed or endorsed by the publisher.
