# Supplementary material for: Trends and Developments in the Detection of Pathogens in Central Nervous System Infections: A Bibliometric Study
Source: Front Cell Infect Microbiol. 2022 Apr 29;12:856845. doi: 10.3389/fcimb.2022.856845 (PMC9100591; doi:10.3389/fcimb.2022.856845)
Supplement: Supplementary file 1 [file DataSheet_1.docx]

Supplementary Figure 1. Supplementary Material

# Supplementary Figures

## Supplementary Figure 1


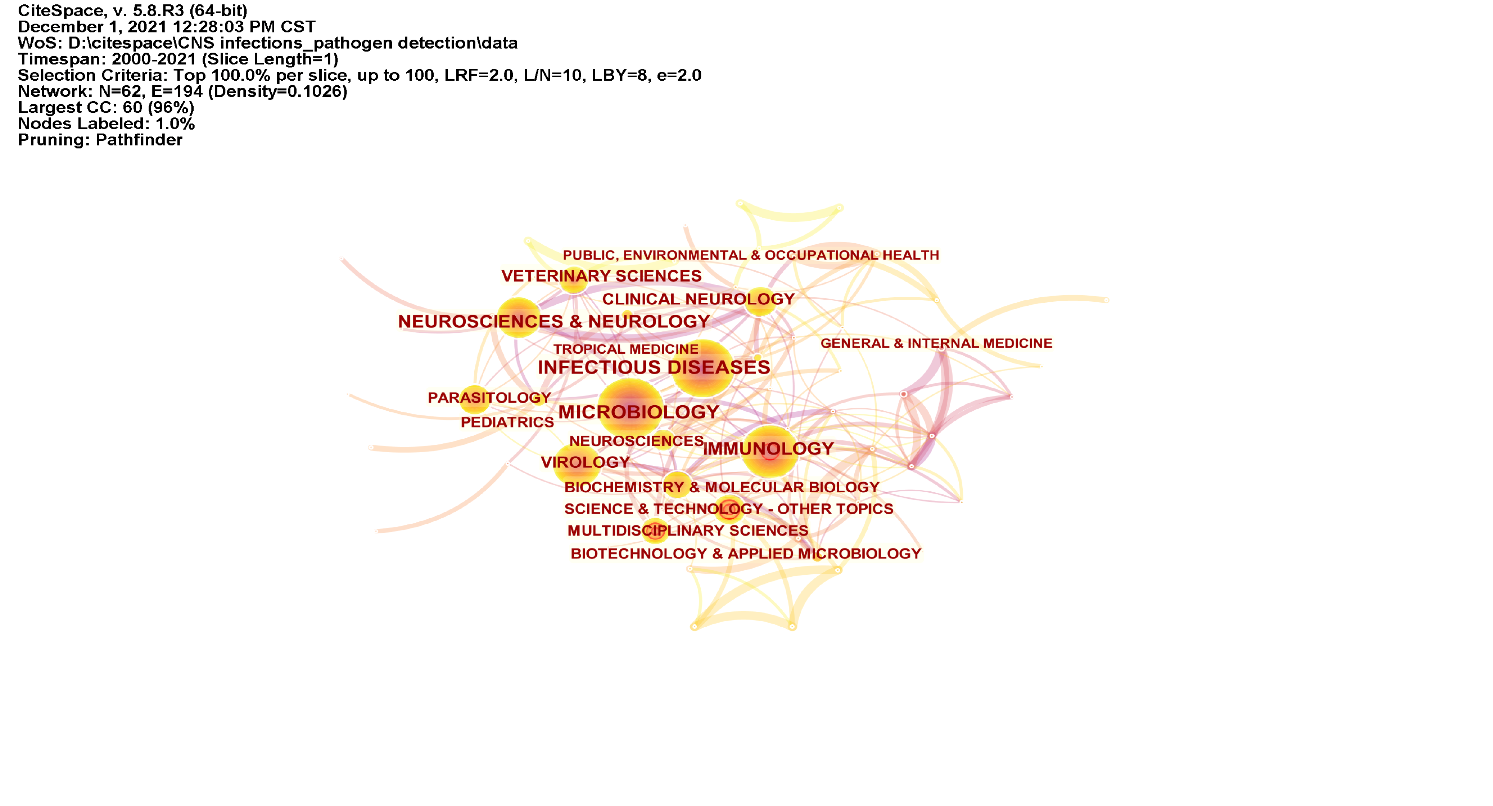


**Supplementary Figure 1.** Disciplines involved in the detection of pathogens for CNS infections, shown as a Pathfinder network of subject categories.

## Supplementary Figure 2


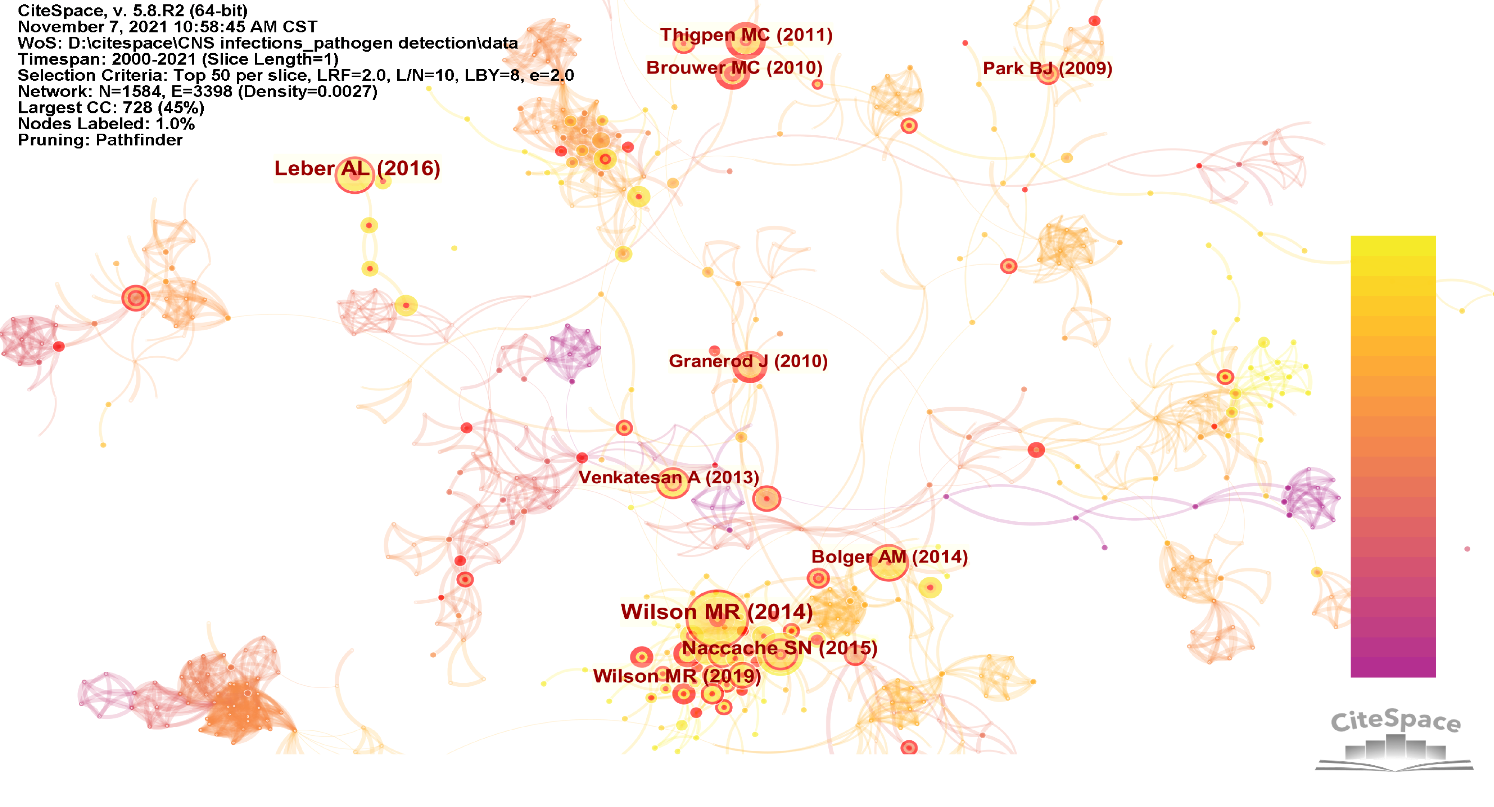


**Supplementary Figure 2.** Clustering visualization of highly cited publications on pathogen detection for CNS infections.

# Supplementary Tables

## 2.1 Supplementary Table 1. Top 10 countries/regions in terms of publication number.

| **Publication number** | **Country** |
| --- | --- |
| 769 | USA. |
| 309 | PEOPLES R CHINA. |
| 146 | ENGLAND. |
| 135 | GERMANY. |
| 102 | FRANCE. |
| 100 | JAPAN. |
| 85 | AUSTRALIA. |
| 75 | CANADA. |
| 70 | SWITZERLAND. |
| 70 | NETHERLANDS. |

## 2.2 Supplementary Table 2. Top 10 institutions regarding publication number.

| **Publication number** | **Institution** | **Country** |
| --- | --- | --- |
| 53 | Ctr Dis Control & Prevent | USA. |
| 48 | Univ Oxford | ENGLAND. |
| 28 | Univ Calif San Francisco | USA. |
| 28 | Johns Hopkins Univ | USA. |
| 26 | Inst Pasteur | France. |
| 26 | NIAID | USA. |
| 24 | Univ Texas Med Branch | USA. |
| 24 | Univ Amsterdam, | NETHERLANDS. |
| 24 | Univ Minnesota, | USA. |
| 23 | Capital Med Univ | PEOPLES R CHINA. |

**2.3 Supplementary Table 3.** Top 10 researchers regarding publication number.

| **Publication number** | Researcher |
| --- | --- |
| 9 | XIN WANG |
| 9 | JENNIFER DIEN BARD |
| 7 | MICHAEL R WILSON |
| 7 | JACOB LORENZOMORALES |
| 6 | JR PERFECT |
| 5 | MARCELO GOTTSCHALK |
| 5 | ROBIN PATEL |
| 5 | TOM SOLOMON |
| 5 | HEIN SPRONG |
| 5 | XAVIER DE LAMBALLERIE |
